# The Key Role of Cyclic Electron Flow in the Recovery of Photosynthesis in the Photobiont during Rehydration of the Lichen *Cladonia stellaris*

**DOI:** 10.3390/plants12234011

**Published:** 2023-11-29

**Authors:** Shuzhi Wang, Wenfeng Li, Rehemanjiang Wufuer, Jia Duo, Liang Pei, Xiangliang Pan

**Affiliations:** 1 National Engineering Technology Research Center for Desert-Oasis Ecological Construction, Xinjiang Institute of Ecology and Geography, Chinese Academy of Sciences, 818 South Beijing Road, Urumqi 830011, China; liwf86@ms.xjb.ac.cn (W.L.); reheman319@ms.xjb.ac.cn (R.W.); duojia2017@ms.xjb.ac.cn (J.D.); peiliang@ms.xjb.ac.cn (L.P.); 2 Xinjiang Key Laboratory of Environmental Pollution and Bioremediation, Xinjiang Institute of Ecology and Geography, Chinese Academy of Sciences, Urumqi 830011, China; 3 Key Laboratory of Microbial Technology for Industrial Pollution Control of Zhejiang Province, College of Environment, Zhejiang University of Technology, Hangzhou 310014, China

**Keywords:** P700^+^ absorbance, chlorophyll *a* fluorescence, photosystem I, photosystem II, cyclic electron flow, non-photochemical quenching, rehydration, lichen

## Abstract

Lichens are poikilohydric organisms and an important part of the ecosystem. They show high desiccation tolerance, but the mechanism of dehydration resistance still needs to be studied. The photosynthesis recovery of the photobiont in rehydrated lichen *Cladonia stellaris* after 11-year desiccation was investigated by simultaneously monitoring both photosystem I and II (PSI and PSII) activities. The responses of the photochemical efficiency and relative electron transport rate (rETR) of PSI and PSII, and the quantum yield of the cyclic electron flow (CEF) were measured using a Dual-PAM-100 system. PSI recovered rapidly, but PSII hardly recovered in *C. stellaris* during rehydration. The maximal photochemical efficiency of PSII (*F*_v_/*F*_m_) was generally very low and reached about just 0.4 during the rehydration. These results indicated that PSII had restored little and was largely inactivated during rehydration. The quantum yield of PSI recovered quickly to almost 0.9 within 4 h and remained constant at nearly 1 thereafter. The results showed that the activation of the CEF in the early stages of rehydration helped the rapid recovery of PSI. The quantum yield of the CEF made up a considerable fraction of the quantum yield of PSI during rehydration. A regulated excess energy dissipation mechanism and non-photochemical quenching (NPQ) also recovered. However, the small extent of the recovery of the NPQ was not enough to dissipate the excess energy during rehydration, which may be responsible for the weak activity of PSII during rehydration. The results indicated that both CEF and NPQ were essential during the rehydration of the photobiont in *C. stellaris*. The methods used in the measurements of chlorophyll *a* fluorescence and P700^+^ absorbance changes in this study provided a speedy and simple way to detect the physiological characteristics of the photobionts of lichen during rehydration. This work improves our understanding of the mechanism behind lichen’s desiccation tolerance.

## 1. Introduction

Lichens are symbiotic organisms composed of lichen-forming fungi (mycobionts) and certain groups of cyanobacteria or green algae (photobionts) [1,2]. Lichens are an important component of the biological soil crusts of the soil surface in most arid and semi-arid regions around the world and are among the most important biotic components of the ecosystems in these areas [3,4]. As the number of climate change-induced drought events increases, agricultural land and food production are vulnerable to future water scarcity [5]. In this context, lichens can still be the guarantee of the maintenance and restoration of ecosystem productivity under extreme climate changes. Photosynthetic organisms, including lichens and their photobionts, which are tolerant to desiccation, have been widely investigated to improve drought tolerance in crop plants [6].

It has been reported that lichens are desiccation-tolerant organisms that can survive in harsh environments where higher plants cannot survive [7,8]. Lichens are often found in a variety of extremely dry habitats such as rock surfaces, the Antarctic cold desert and hot deserts [9,10]. In these habitats, the water supply is not delivered by rain but rather in the form of fog, dew or humidity [8,11]. Lichens are poikilohydrous and exposed to repeated desiccation/rehydration cycles [9]. Consequently, lichens may be exposed to higher levels of solar radiation and desiccation, and most lichens can tolerate desiccation and remain viable for months [12]. During the desiccation/rehydration cycles, lichens and their photobionts use a variety of ways to cope with various stresses. These stresses include osmotic stress and oxidative stress, which are related to sudden changes in water availability [13]. Dehydrated lichens can restore their photosynthetic activity after wetting [14]. The microalgae isolated from a Mediterranean fruticose epiphytic lichen that adapted to xeric habitats, i.e., *Trebouxia* sp. (TR9), could recover their photosynthetic activity after desiccation for 1, 2 and 3 months [15].

The understanding of desiccation tolerance of lichens and lichen-forming algae was reviewed in a previous study, which showed the constitutive mechanisms and the induction of protection mechanisms in the desiccation tolerance of lichens [2]. However, the summarized data revealed that the knowledge about desiccation tolerance mechanisms in lichens was much scarcer than in other organisms such as bryophytes or vascular resurrection plants. Some previous studies showed that lichens can become photosynthetically active after rehydration with water vapor alone or with liquid water [11,16,17,18]. Those studies mainly discussed the recovery of the activity of photosystem II (II), which was often tested by measuring chlorophyll *a* fluorescence [19,20]. Some dehydration and rehydration experiments were carried out with algae isolated from lichens [6,21]. The findings with *Trebouxia*, the most abundant chlorophytic photobiont in lichen, indicated that the slow-dried and rapid-dried algae showed significant differences in the recovery of photosynthetic activity. Slow-dried algae restored PSII electron transport to a higher value after rehydration. Measurement of the absorbance change in P700 of *Trebouxia* showed that desiccation did not affect PSI functionality [21]. However, only a small number of species of lichens and bryophytes have actually been tested for desiccation tolerance, and the mechanisms for testing desiccation tolerance are still limited [11]. Additionally, the response of photochemical efficiency and the relative electron transport rate (rETR) of photosystem I (PSI) during the desiccation and rehydration of lichens are still unclear. Little is known about the differences between the recovery potential of PSII and PSI in the rehydration process and the underlying mechanisms.

It was proposed that the cyclic electron flow (CEF) around PSI functioned in adaptation to environmental stress in higher plants [22,23]. Gao and Wang (2012) found that the CEF in *Porphyra yezoensis* (Bangiales, Rhodophyta) played a significant physiological role during desiccation and rehydration [24]. Beckett et al. (2023) suggested that dual PAM could effectively measure alternative electron flows in lichen photobionts [25], and thereby it could be used to study how lichens coped with the desiccation and rehydration conditions. The results showed that the CEF appeared to be much higher in lichen photobionts than angiosperms [25]. However, whether the CEF is activated during rehydration after desiccation and the physiological role of CEF in the photobiont of lichen remain unclear.

Although the existing literature shows that most lichens can tolerate desiccation and remain viable for months [12], only a small number of species of lichens have actually been tested for desiccation tolerance. The time limit for lichens to withstand dehydration was not well studied. This work will explore whether lichens have the possibility to restore their photosynthetic activity after long-term dehydration for more than 10 years. In our preliminary experiments, we found that *Cladonia* species like *Cladonia stellaris* could tolerate desiccation for months. We postulate that symbiotic algae can react quickly in the process of rehydration from drought to restore the physiological activity. The rapid recovery of photosynthetic organisms can promote the rehydration of lichens and provide their fungal energy substances, which plays an important role. The objective of this study is to test the recovery of the activities of the photosystems and the physiological role of CEF in the photobiont of lichen during rehydration after long-term desiccation. The thalli of *Cladonia stellaris* (Opiz) Pouzar & Vězda, which had been kept dry for 11 years, were used to perform the rehydration experiment. The response of the photochemical efficiencies and rETRs of PSI and PSII of the photobiont were measured during rehydration with the help of a Dual-PAM-100 system. The CEF was also measured to detect whether it was activated and to reveal its physiological role during rehydration. The results showed that PSI recovered rapidly during rehydration, but PSII hardly recovered in the experiment. The quick activation of the CEF played an important role in the recovery of the activity of PSI.

## 2. Results

### 2.1. Scanning Electron Microscopy (SEM) Observation

**Figure 1:**
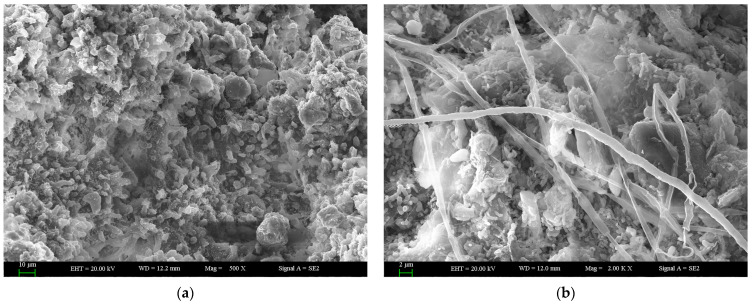

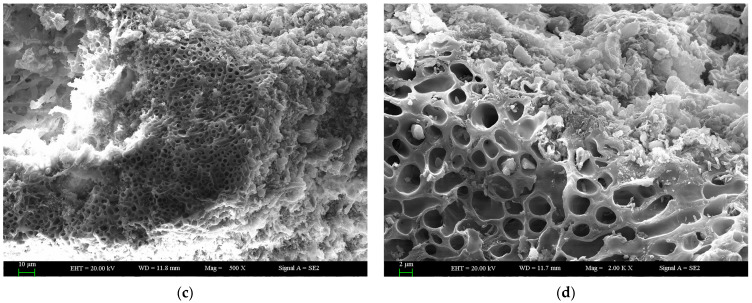
SEM images of the microstructure of thalli of *C. stellaris*. (**a**) Image of the surface of thalli; (**b**) the mycobionts or fungal hyphae on the surface of the thalli; (**c**) the structure of the surface and a cross-section; (**d**) an enlarged image of the cross-section.

SEM images showed the microstructure of the surface and cross-section of the thalli of *C. stellaris* after rehydration for 21 h (Figure 1). The structure of the thalli was incompact, and the main part of the thalli was constructed by the mycobiont or fungal hyphae. Figure 1a displays the overall morphology of the surface of a lichen thallus (bar = 10 μm). The distribution of fungal hyphae and photobionts is shown on the surface of the thalli (Figure 1a,b). A cross-section of the thalli is also shown, where many pores are located (Figure 1c). The structure of the pores can be seen more clearly in Figure 1d. The cortex and medulla of the thalli are presented (Figure 1c,d). In a cross-section of the thalli, the internal surfaces of the lumens of fungal hyphae are shown (Figure 1d, bar = 2 μm).

### 2.2. Maximal Photochemical Efficiency of PSII (F_v_/F_m_)

**Figure 2:**
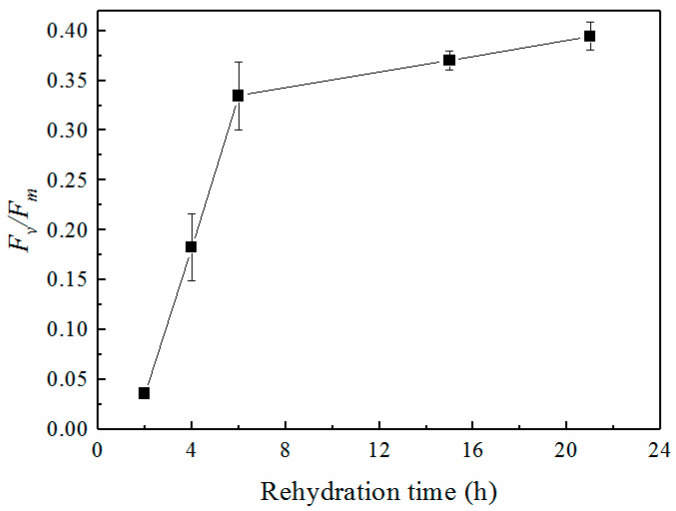
Maximal photochemical efficiency of PSII (*F*_v_/*F*_m_) during rehydration. Data were detected after 20 min dark-adaptation during each measurement (n = 4).

Pieces of thalli of *C. stellaris* were rehydrated to test the recovery of photosynthetic activity. *F*_v_/*F*_m_ increased with the rehydration time (Figure 2). After rehydration for 2, 4 and 6 h, *F*_v_/*F*_m_ reached 0.04, 0.18 and 0.33, respectively. *F*_v_/*F*_m_ recovered rapidly during the first 6 h and increased slightly thereafter. *F*_v_/*F*_m_ was generally very low (below 0.4) over the entire experimental time, which was much lower than that of other photosynthetic organisms.

### 2.3. Quantum Yields of Two Photosystems and CEF during Rehydration

**Figure 3:**
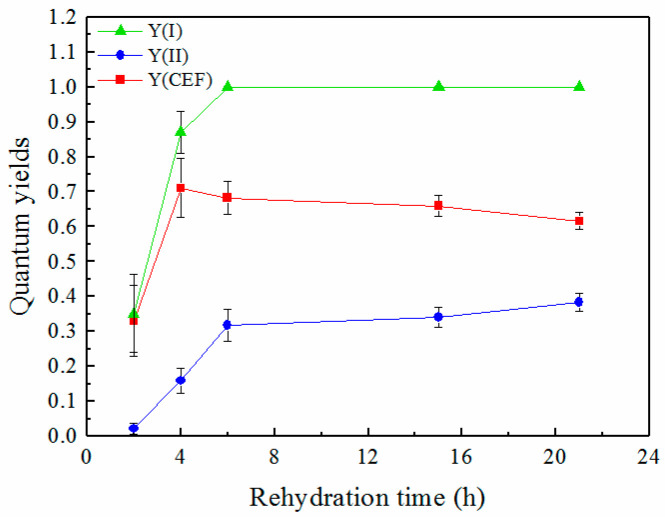
Quantum yields of two photosystems and CEF during rehydration. Data were detected at the beginning of the rapid light curve mode, where intensity of the actinic light was 30 μmol m^−2^ s^−1^, and the actinic light lasted for 30 s (n = 4).

The values of Y(I), Y(II) and Y(CEF) at the beginning of the rapid light curve mode (RLC mode) (where the PAR intensity of the actinic light was 30 μmol m^−2^ s^−1^) changed differently during rehydration (Figure 3). The measurements in RLC mode were conducted with an actinic light with increasing intensity (30, 37, 46, 77, 119, 150, 240, 363, 555 and 849 μmol m^−2^ s^−1^). A saturating pulse was applied after each period of actinic light to determine the maximum fluorescence and P700^+^ signals. Parameters such as quantum yields and electron transport rates (ETRs) are calculated based on measurements of these signals. Y(I) recovered quickly to almost 0.9 within 4 h and remained constant at nearly 1 thereafter. Y(II) increased a little and reached about 0.4 at 21 h. Y(CEF) rapidly increased during the first 4 h of rehydration and then decreased a little with the rehydration time.

### 2.4. RLCs of Y(I), Y(II) and Y(CEF)

**Figure 4:**
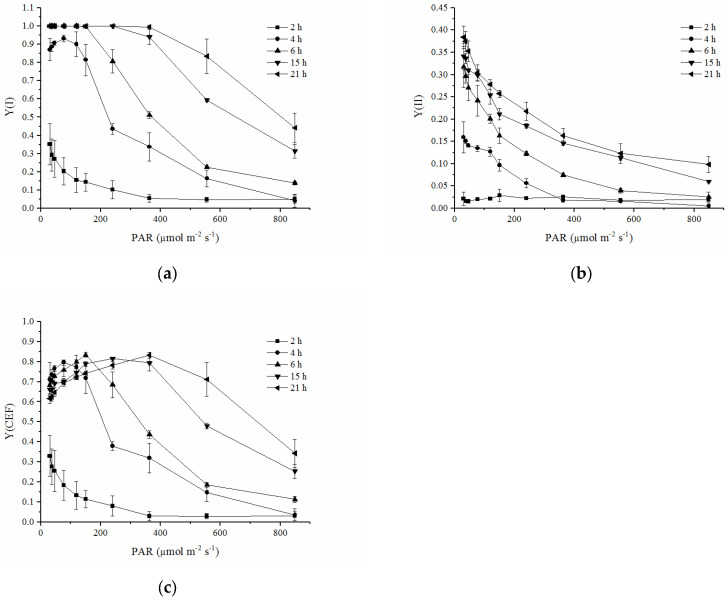
Rapid light curves (RLCs) of quantum yields during rehydration. (**a**) RLCs of Y(I); (**b**) RLCs of Y(II); (**c**) RLCs of Y(CEF). Data were detected from the RLC mode, where PAR increased from 30 to 849 μmol m^−2^ s^−1^ (n = 4).

After the thalli were rehydrated for 4 h, Y(I) was almost saturated at the beginning PAR intensity of the RLC and decreased with an increasing PAR intensity. When the lichen was rehydrated for 6 h or longer, Y(I) was able to keep steady at low PAR intensities. Generally, the RLCs of Y(I) shifted upward remarkably with the rehydrating time (Figure 4a). The RLCs of Y(II) also moved upward with the rehydration time but were much lower than the RLCs of Y(I) at the same rehydration time (Figure 4b). Y(II) drastically decreased with an increasing PAR intensity. Y(CEF) decreased with an increasing PAR intensity after the lichen was rehydrated for 2 h. However, as the rehydration time was prolonged, Y(CEF) increased with an increasing PAR intensity in the range of lower intensity (less than 363 μmol m^−2^ s^−1^ after rehydration for more than 15 h) and then decreased with an increasing PAR intensity in the higher PAR range (Figure 4c). Similarly, the RLCs of Y(CEF) showed an upward shift as the rehydration time was prolonged.

### 2.5. RLCs of rETR(I) and rETR(II)

**Figure 5:**
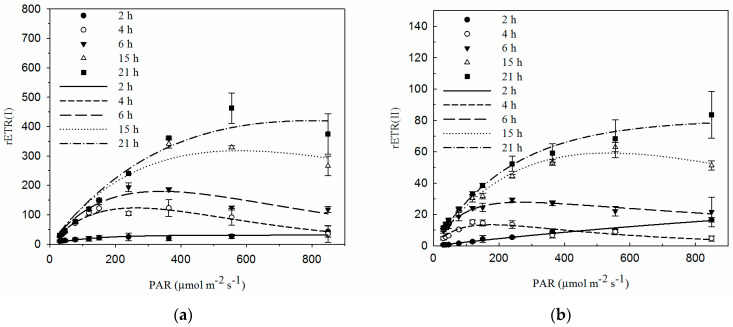
Rapid light curves (RLCs) of the relative electron transport rates (rETRs) of PSI and PSII during rehydration. (**a**) RLCs of the rETR(I). (**b**) RLCs of the rETR(II). Data were detected from the RLC mode, where PAR increased from 30 to 849 μmol m^−2^ s^−1^. The fitting curves are also shown in the graphs (n = 4).

ETRs are expressed as rETRs in the present study, and the equation to calculate ETRs was modified due to a lack of uncertain coefficients such as an absorption factor. The calculation of rETRs is shown in Materials and Methods (Section 4.6). With the values of the rETR(I) and the rETR(II), an overestimation of the ETRs may occur. But the results could still indicate a variation trend in ETRs during rehydration. Both the amplitude of the RLCs of the rETR(I) and the rETR(II) increased during rehydration (Figure 5). At each rehydration time, the rETR(I) was higher than the rETR(II). When the rehydration time was less than 6 h, the rETR(I) and the rETR(II) increased at low light intensities and then decreased at high light intensities. At 15 and 21 h after rehydration, the rETR(I) and the rETR(II) could be maintained at relatively high values at higher light intensities.

The descriptive RLC parameters are shown in Table 1. Since the optical properties and the absorption factor were difficult to measure for the non-flat thalli as shown in references [26,27], the equation to calculate ETRs in the present study did not contain coefficients such as the absorption factor used in the common equation. Due to the calculation of rETRs and the resultant overestimation of ETRs, these descriptive RLC parameters were relative values to show the variation trend. In particular, *α,* the initial slope of the RLC of the rETR, could only reflect the relative size between different measurements, but showed no actual significance because of the fact that photochemical efficiency values higher than 1.0 were physically impossible. *E*_k_, *α,* and the rETR_max_ of the rETR(I) were higher than those of the rETR(II) during rehydration. All these descriptive parameters increased with the rehydration time. r*E*_k_, *α* and the rETR_max_ of PSI increased significantly as the rehydration time was prolonged and recovered faster than those of PSII (*p* < 0.05). r*E*_k_ and the rETR_max_ of PSII showed no significant increase during the first 6 h, with values lower than those of PSI.

### 2.6. RLCs of Non-Photochemical Quenching

**Figure 6:**
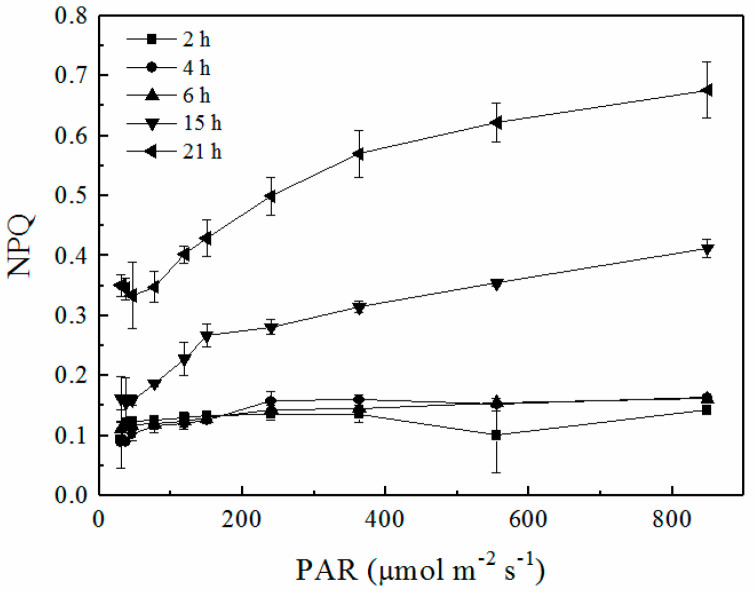
Rapid light curves (RLCs) of non-photochemical quenching (NPQ) during rehydration. Data were detected from the RLC mode, where PAR increased from 30 to 849 μmol m^−2^ s^−1^ (n = 4).

The NPQ did not increase significantly with an increasing PAR intensity during the first 6 h of rehydration (Figure 6). Significant increases in NPQ with an increasing PAR intensity were observed 15 h or more after rehydration. The NPQ at 21 h was nearly double than that at 15 h. Generally, the NPQ was still very low during rehydration compared to that of other lichens [2,30].

## 3. Discussion

The desiccation tolerance and rehydration of lichen have been widely studied. Enhancement of the understanding of the drought resistance mechanisms of lichens can contribute to ensuring agricultural production and maintaining ecosystem stability under climate change-induced drought events. It was confirmed that the genes related to photosynthesis are strongly overexpressed in response to desiccation in lichen-forming chlorophyte [2], indicating the important role of photosynthesis in the desiccation resistance of lichen. Both chlorophyll *a* fluorescence and P700^+^ absorbance changes in the photobiont in the lichen *C. stellaris* were monitored to examine the activities of PSII and PSI during rehydration in the present study.

Chlorophyll *a* fluorescence was used as a powerful tool to assess the state of the photosynthetic apparatus in response to different environmental conditions or stresses [31,32]. P700^+^ absorbance changes were monitored to determine the quantum yield of PSI [33,34]. Some studies have applied these techniques to study the mechanisms in the physiology of the isolated photobionts from lichens in response to desiccation and light stress [25,35]. To our knowledge, this is the first study that simultaneously detected the responses of the quantum yields and ETRs of PSI and PSII of the photobiont components of the lichen during rehydration after long-term desiccation and has revealed the important role of the CEF in the photobiont of lichen during rehydration. Although the CEF could explain the imbalance in PSI/PSII photochemical efficiency, the result is indirect—neither the fluorescence nor the absorbance transients are direct measures of the absolute photochemical quantum yield. This study is preliminary. As in some other studies, the measurement of quantum yields and electron transport rates in two photosystems, as well as the estimation of the CEF, could still play a valuable role in explaining the response of photosynthetic organisms to environmental stresses although the estimation was not entirely accurate [36,37]. The applications of Chlorophyll *a* fluorescence and P700^+^ absorbance changes could provide us a speedy and simple way to detect the physiological characteristics of the photobionts of lichen during rehydration.

It was found that lichens could rapidly change their physiological activity, such as respiration and photosynthesis [7,19], in response to rapid changes in water content to equilibrium with the water status of the environment [7,11]. In the lichen thalli, the mycobionts form a protective “matrix” that provides the living space and structural foundation for the photobionts. According to the observation of the microstructure of the thallus, the intervals and pores in the thalli were conducive to water absorption, which provided a favorable structural basis for the recovery of physiological activity during the rehydration of lichens (Figure 1). This porous structure has also been found in other studies about the microstructure of lichens. The internal surfaces of the lumens of fungal hyphae were similar to previous studies [38,39]. The microstructure of the thallus benefits the photosynthesis and growth of symbiotic algae, by providing the convenience for water absorption and gas exchange. The photobionts located in the thalli sensed water quickly and recovered their physiological activities, such as photosynthesis, during rehydration. Some previous studies showed that lichens can become photosynthetically active after rehydration, indicated by the recovery of the ETR and the *F*_v_/*F*_m_ of PSII [19,40]. Green et al. (2002) found that the photon efficiency and ETR of PSII of the photobiont components of a photosymbiodeme could become activated after rehydration under normal conditions [16]. Calatayud et al. (1997) found that the recovery of *F*_v_/*F*_m_ of five lichens during rehydration was dependent on species: *F*_v_/*F*_m_ recovered to 0.745, 0.659, 0.732 and 0.694 for *Parmelia acetabulum*, *Ramalina farinacea*, *Pseudevernia furfuracea* and *Evernia prunastri*, respectively [19]. Gauslaa and Solhaug (2000) also found that the *F*_v_/*F*_m_ of lichen could recover to its optimal high value in the field or in the laboratory [40]. The *F*_v_/*F*_m_ of *C. stellaris* increased to around 0.4 (Figure 2) in the present study. Such a low value of *F*_v_/*F*_m_ might indicate damage to PSII or the inactivated state of PSII during rehydration or long-term desiccation. These results were different from some studies that showed the fast recovery of PSII upon rehydration [17], mainly due to differences between long-term desiccation and short-term desiccation. Prolonged drying and dehydration cause greater damage to the structure and functional recovery of PSII than short-term desiccation. This is in line with some previous studies that showed that only a prolonged stay in the desiccated state caused significant degradation of chlorophyll [41,42]. The desiccation tolerance is species-specific and depends on the speed, intensity and duration of water loss during dehydration [6].

In contrast to PSII, PSI recovered rapidly during rehydration (Figure 3). Y(I) reached almost 0.9 within 4 h and stayed at a high value thereafter (Figure 3). These findings were consistent with the results in the thalli of the lichen *Hypogymnia physodes* during their rehydration [43]. Rehydration of air-dried *H. physodes* caused the gradual restoration of photosynthetic activities in both PSII and PSI, but the recovery was faster in PSI [43]. A lessened recovery of PSII could also be confirmed by low Y(II) (Figure 3 and Figure 4b) during rehydration. PSII has been demonstrated to be very sensitive to various environmental stresses, such as heavy metals, high or low temperatures, and desiccation [24,44,45]. It was proposed that the relatively slow recovery of PSII may be due to the need for more elaborate processes, such as the photoactivation of Mn clusters in the water-oxidizing complex [43]. The faster recovery of PSI activity was also indicated by the significant increase in the rETR(I) (Figure 5a) and its RLC parameters (*α*, rETR_max_ and r*E*_k_) (Table 1).

The RLCs and the descriptive parameters of the RLCs of the rETR(I) and the rETR(II) provided more information on the responses of electron transport in PSI and PSII to increasing irradiation. Commonly, the equations for ETRs typically include a coefficient of 0.5 and an additional coefficient of 0.84. These equations are based on the assumption that the absorbed quanta are distributed between the two photosystems. Some measurements like the pigment composition and contents were not conducted in this study, and the absorption coefficients were not known. As a consequence, ETRs are expressed as rETRs in the present study. This kind of estimation of ETRs was used in some previous studies where optical properties of the samples were hard to estimate, and the absorption factor and the photosystem stoichiometry were not known [26,27]. Nitschke et al. (2012) applied the calculation of rETRs and pointed out that it was not intended to interpret rETRs as an estimation of primary production, and the rETRs of different species could not be compared. But the relative changes in the values of one species allowed an estimation of changes in its photo-response capacity [26]. Similar to previous studies [26,27], the calculation of rETRs provided relative values that were overestimated in the present study. Even though, as we aimed at detecting and comparing the response of two photosystems and the potential role of the CEF during rehydration, the trends of changes derived from the present results were meaningful. The relative changes in the photosynthetic performance of the photobiont calculated as rETR allowed an estimation of its photo-response capacity during rehydration. The lower values and slower recovery of the rETR(II) (Figure 5b) and the lower rETR(II) RLC parameters (Table 1) compared to those of the rETR(I) further confirmed that PSI recovered faster and to a better state than PSII (Figure 5 and Table 1). Similarly, Gao and Wang (2012) found that PSI activity in desiccated blades of *Porphyra yezoensis* (Rhodophyta) recovered faster than PSII activity during rehydration [24].

The activation of the CEF and a high value of the quantum yield of the CEF at the early stages significantly contributed to the rapid recovery of PSI. Previous research indicated that the electrons generated through linear electron transport involving PSII were not sufficient to reduce all PSI centers during the rehydration of the thalli of the lichen *H. physodes*, and the researchers proposed that some PSI units received electrons via alternative routes. The relatively slow electron flow to P700^+^ has been suggested to proceed from reductants localized in the chloroplast stroma [43]. Besides some data suggesting the activity of a pseudo cyclic electron flow (PCEF) mediated by a class of enzymes called flavodiiron proteins (FLVs) in the common lichen photobiont *Trebouxia*, there have been few studies on CEF in lichens [25,46]. An increase in the CEF was detected in the isolated photobiont of the lichen *Pleopsidium chlorophanum* after 7-day desiccation and played a protective role in the photosynthetic apparatus, and the risk of photoinhibition of PSI of the photobiont of *Fulgensia bracteata* might be minimized by inhibition of the linear electron flow and the increased CEF [35]. The present study found that Y(CEF) made up a considerable fraction of Y(I) during rehydration. Gao and Wang (2012) suggested that the CEF in *P. yezoensis* played a significant physiological role during the recovery after rehydration of desiccated algae [24]. We also found a similar phenomenon in the present study, indicating that the activation of the CEF is essential for the recovery and protection of the activity of PSI.

The CEF was considered to contribute to the formation of the trans-thylakoid membrane proton gradient (ΔpH), which induces the synthesis of ATP [47,48]. More ATP could be used to promote the recovery of the photosynthetic apparatus and the synthesis of relevant enzymes for scavenging ROS that may accumulate in the lichen during dehydration [11,49]. While results from the lichen *H. physodes* showed that the major mechanism to protect PSI in dehydrated lichens was the recombination rather than acceleration of the rates of the CEF that led to the accumulation of oxidized P700 [43]. However, stimulation of the CEF and the ΔpH generated by CEF could still play a part in preventing the release of intracellular free radicals and regulating lipid peroxidation, and thus play important roles in protecting lichens from damage.

The ΔpH generated by the activation of CEF under environmental stress functions to regulate light harvesting by inducing high-energy state quenching and relates to xanthophyll cycle-dependent NPQ [23,50]. The recovery of the regulated excess energy dissipation mechanism NPQ was also observed in the present study (Figure 6). The NPQ increased during rehydration and began to respond to increasing light intensity to regulate energy dissipation after 15 h. However, the NPQ was not effectively recovered in the present study in comparison with the values of NPQ for lichens in an optimal state or during rehydration after desiccation [19,20]. The majority of recent studies on mechanisms of lichen desiccation tolerance have focused on the scavenging of ROS. The relationships between ROS, NPQ and desiccation tolerance in lichens have been reviewed in previous studies [2,7]. The NPQ process acted as an important photoprotective mechanism during the desiccation and rehydration of lichen [30]. NPQ released excess light energy from PSII, thereby preventing oxidative stress [35]. The inactive PSII did not show strong NPQ in this study. We propose that the lack of NPQ affected the activity of PSII during rehydration.

## 4. Materials and Methods

### 4.1. Rehydration of Cladonia stellaris

*C. stellaris* (Opiz) Pouzar & Vězda was collected in the air-dried state from Aletai, Xinjiang, China. It contains green alga *Trebouxia* (Chlorophyta) as photobionts. The thalli of *C. stellaris* were kept dry for 11 years at room temperature with relative humidity less than 10%. The thalli were washed and rehydrated with deionized water and then placed on wet filter papers in culture dishes under continuous fluorescent white light (30 μmol photons m^−2^ s^−1^) at 25 °C [51]. The thalli were kept wet in water vapor for the recovery experiments [52].

### 4.2. Scanning Electron Microscopy (SEM)

After rehydration for 21 h, the thalli of *C. stellaris* used for SEM observation were washed and dehydrated with 70% ethanol solution. Then, the thalli were dried and sputter-coated with gold. The SEM observation was carried out with a scanning electron microscope (Zeiss Super 55VP, Hamburg, Germany) at an acceleration voltage of 20 kV.

### 4.3. Application of the Dual-PAM-100 System

The activities of photosystems and CEF in the photobiont of *C. stellaris* during rehydration were probed using chlorophyll *a* fluorescence and P700^+^ absorbance, which were measured simultaneously using a Dual-PAM-100 system (Heinz Walz GmbH, Effeltrich, Germany) [47]. Then, 2, 4, 6, 15 and 21 h after onset of rehydration, the thallus was sandwiched between the emitter head and detector head of the device. The measurements were performed using the automated Dual-PAM induction program. The thalli were dark-adapted for 20 min before the tests. The minimal fluorescence after dark adaptation (*F*_0_) was detected using a measuring light at low intensity. A 300 ms saturating pulse of 10,000 μmol photons m^−2^ s^−1^ was then applied to detect the maximum fluorescence after dark adaptation (*F*_m_). The maximal photochemical efficiency of photosystem II (PSII) was calculated as

*F*_v_/*F*_m_ = (*F*_m_ − *F*_0_)/*F*_m_ (1)

After the determination of *F*_0_ and *F*_m_, the maximal change in P700^+^ signal (*P*_m_) was determined through the application of a saturation pulse after 10 s far-red pre-illumination [53].

### 4.4. Measurement of the Rapid Light Response Curves (RLCs)

After the determination of *F*_0_, *F*_m_ and *P*_m_, RLCs were recorded in the rapid light curve mode (RLC mode). RLCs were used to reflect the photosynthetic performance in response to increasing light intensity. In comparison to normal light curves (LCs), RLCs normally measure the effective quantum yield or electron transport rate as a function of irradiance with increasing intensity [29]. During RLC, photosynthetically available radiation (PAR) is provided with increasing intensity, and each light increment lasts for shorter time than normal LC. RLC takes less time to test the activity of a photosynthetic apparatus and has advantages in detecting the response of photosynthetic organisms to changing environments [54]. RLCs were measured to describe the response of quantum yields and rETRs of photosystems, and the response of CEF to increasing light intensity during rehydration. The measurements in RLC mode were conducted with an actinic light applied at each PAR for 30 s with increasing intensity (30, 37, 46, 77, 119, 150, 240, 363, 555 and 849 μmol m^−2^ s^−1^). A saturating pulse was applied after each period of actinic light to determine the maximum fluorescence signal (*F*_m_′) and maximum P700^+^ signal (*P*_m_′) under the actinic light. RLCs with saturation pulse analysis were based on the determined *F*_0_, *F*_m_ and *P*_m_.

### 4.5. Quantum Yields of the Photosystems and the CEF

The quantum yields of PSI and PSII were detected using saturating pulses after each PAR in RLC mode. The effective photochemical quantum yield of PSII (Y(II)) was calculated according to the method of Kramer et al. (2004) [55], which can be transformed into the following simpler equation:

Y(II) = (*F*_m_′ − *F*)/*F*_m_′ (2)

where *F* is the steady state fluorescence [56].

The effective photochemical quantum yield of PSI (Y(I)) was calculated according to Klughammer and Schreiber (2008) [34]:

Y(I) = (*P*_m_′ − *P*)/*P*_m_ (3)

where *P* (the P700^+^ signal) was recorded just before a saturation pulse [34].

The quantum yield of the CEF was calculated from Y(I) and Y(II) referring to Huang et al. (2010) [47]:

Y(CEF) = Y(I) − Y(II)(4)

### 4.6. RLCs of Relative Electron Transport Rates in PSI and PSII

Since the optical properties of the thalli were not estimated and the absorption factor was difficult to measure for the non-flat thalli, ETRs were expressed as rETRs and the common calculation of ETRs was modified [27]. So, we used the terms rETR(I) and rETR(II) to reflect relative electron transport rates in PSI and PSII. rETRs were defined and calculated according to Nitschke et al. (2012) [26] and Saroussi and Beer (2007) [27], without the introduction of uncertain coefficients such as the absorption factor used in the common equation to calculate ETRs:

rETR(I) = Y(I) × PAR(5)

rETR(II) = Y(II) × PAR(6)

Although the values of the rETR(I) and rETR(II) led to overestimation of ETRs, the results derived from the calculation could still meet our need to reveal the characteristics of physiological changes and the variation trend in ETRs during rehydration in the present study. Furthermore, descriptive parameters of the rETR(I) and rETR(II) were derived by fitting the RLCs to the a double exponential decay function as described in Platt et al. (1980) [28] and Ralph and Gademann (2005) [29]: *α* is the initial slope of the RLC of rETR, reflecting the photochemical efficiency [28,57]. rETR_max_ is the maximum relative electron transport rate. According to the calculation, *E*_k_ values were termed relative *E*_k_ (r*E*_k_) in the present study. r*E*_k_, the sub-saturation irradiance, is the index of light adaptation of a photosystem and calculated as rETR_max_/*α* [28,58].

### 4.7. Energy Dissipation by Non-Photochemical Quenching

Non-photochemical quenching (NPQ) was analyzed to detect whether the regulated energy dissipation mechanism was enhanced to protect the photosystems from reactive oxygen species (ROS) that may accumulate during dehydration and cause damage to the physiological apparatus [11,49]. The NPQ was calculated by referring to Bilger and Björkman (1990) [59]:

NPQ = (*F*_m_/*F*_m_′) − 1 (7)

### 4.8. Statistics

Each measurement was replicated four times. Error bars in all figures represent standard deviation (S.D.). Analysis of variance (one-way ANOVA) and Duncan’s multiple range test were used to determine the significant difference between the data collected at different rehydration times.

## 5. Conclusions

The application of chlorophyll *a* fluorescence and P700^+^ absorbance changes in this study provided a speedy and simple way to detect the physiological characteristics of the photobionts of lichens. This study examined the physiological characteristics of lichen as it rehydrated and restored photosynthetic activity, and explored the sequence of events during the photosynthetic recovery. In summary, the results showed that PSI recovered rapidly, but PSII hardly recovered in *C. stellaris* during rehydration after 11 years of desiccation. Low *F*_v_/*F*_m_ and Y(II) during rehydration implied damage to or inactivation of PSII. PSII was a vulnerable system that hardly recovered or recovered slowly during rehydration. Through the activation of the CEF during rehydration, we speculated that the CEF was essential for the recovery and protection of the activity of PSI and the desiccation tolerance of lichens. The lack of effective NPQ might be responsible for the weak recovery of the activity of PSII during rehydration.

## Figures and Tables

**Table 1:** Descriptive parameters of the rapid light curves (RLCs) of the rETR(I) and the rETR(II). *α*, initial slope of the RLC of rETR; rETR_max_, maximum relative electron transport rate; r*E*_k_, sub-saturation irradiance. Descriptive parameters of the RLCs of the rETR(I) and the rETR(II) were derived by fitting the RLCs to a double exponential decay function as described by Platt et al. (1980) [28] and Ralph and Gademann (2005) [29]. The measurements were carried out after the thalli of the lichen had been rehydrated for 2, 4, 6, 15, and 21 h. Data are means ± S.D. (n = 4), and data followed by different letters in the same column are significantly different (*p* < 0.05). Note: These descriptive RLC parameters are relative values to show the variation trend. In particular, since photochemical efficiency values higher than 1.0 were physically impossible, α could only reflect the relative size between different measurements, but showed no actual significance.

Rehydration Time (h)	Parameters of the Rapid Light Curves of rETR(I)			Parameters of the Rapid Light Curves of rETR(II)		
	*α* (e^−^ Photon^−1^)	rETR_max_ (μmol e^−^ m^−2^ s^−1^)	r*E*_k_ (μmol Photon m^−2^ s^−1^)	*α* (e^−^ Photon^−1^)	rETR_max_ (μmol e^−^ m^−2^ s^−1^)	r*E*_k_ (μmol Photon m^−2^ s^−1^)
2	0.25 ± 0.14 ^a^	33.86 ± 15.10 ^a^	169.26 ± 110.66 ^a^	0.02 ± 0.01 ^a^	7.37 ± 1.17 ^a^	301.87 ± 52.87 ^a^
4	1.32 ± 0.06 ^b^	234.86 ± 43.03 ^b^	177.19 ± 24.35 ^a^	0.24 ± 0.01 ^b^	26.32 ± 10.31 ^a^	112.31 ± 45.72 ^a^
6	1.47 ± 0.04 ^c^	526.87 ± 54.75 ^c^	358.55 ± 45.50 ^b^	0.37 ± 0.03 ^d^	41.09 ± 19.96 ^a^	112.41 ± 60.75 ^a^
15	1.49 ± 0.07 ^c^	1169.89 ± 135.04 ^d^	787.25 ± 86.01 ^c^	0.32 ± 0.01 ^c^	185.79 ± 75.6 ^b^	584.30 ± 253.63 ^b^
21	1.44 ± 0.06 ^c^	1726.43 ± 175.50 ^e^	1197.14 ± 137.53 ^d^	0.35 ± 0.01 ^cd^	206.51 ± 54.94 ^b^	601.41 ± 179.58 ^b^

## Data Availability

The datasets used or analyzed during the current study are available from the corresponding author on reasonable request.
